# Treatment of Fatty Liver Disease: The Present and the Future

**DOI:** 10.7759/cureus.12713

**Published:** 2021-01-15

**Authors:** Sruthi Priyavadhana Ramanan, Mohamed Wael F Mohamed, Su Sandi Aung, Ibrahim Sange, Pousette Hamid

**Affiliations:** 1 Medicine/Surgery, Saveetha Medical College, Chennai, IND; 2 Neurology, California Institute of Behavioral Neurosciences & Psychology, Fairfield, USA; 3 Neurological Surgery, Royal London Hospital, London, GBR; 4 Neurosciences, California Institute of Behavioral Neurosciences & Psychology, Fairfield, USA; 5 Medicine and Surgery, University of Medicine 1, Yangon, MMR; 6 Medicine, KJ Somaiya Medical College, Mumbai, IND

**Keywords:** fatty liver disease, non alcoholic fatty liver disease, drugs for nash, therapeutic targets for nash, molecular targets for nash, genetic targets for nash

## Abstract

Non-alcoholic fatty liver disease (NAFLD) progressing to non-alcoholic steatohepatitis (NASH), cirrhosis, end-stage liver disease (ESRD), and hepatocellular carcinoma (HCC) is emerging as a global epidemic. Obesity, diabetes, and metabolic syndrome are some of the leading risk factors for NAFLD. The most prevalent treatment to stop the progression is aimed at dietary modification and lifestyle changes. Bariatric surgery is indicated for patients with morbid obesity with NAFLD. The progression of NAFLD to NASH and HCC can be arrested at various stages of pathogenesis by the already prevalent drugs and the emerging newer molecular and genetic targets. This review article analyzed various preclinical animal trials and clinical trials and has summarized various groups of drugs that can be life-altering in patients diagnosed with NAFLD. This study also discusses the obstacles in taking these clinical trials to bedside treatment.

## Introduction and background

Non-alcoholic fatty liver disease (NAFLD) is an emerging global epidemic with a prevalence of 25% worldwide [1]. NAFLD consists of varying liver pathology ranging from simple steatosis to ballooning degeneration, inflammation, and fibrosis which are collectively termed as non-alcoholic steatohepatitis (NASH) [2]. NASH has a propensity to progress to liver cirrhosis leading to end-stage liver disease (ESRD) and hepatocellular carcinoma (HCC). This in turn leads to an increase in the rate of a liver transplant due to non-alcoholic liver disease. A landmark study conducted in 2018 suggested that the burden of end-stage liver disease due to NAFLD will be doubled in number by the year 2030 [3]. NASH is frequently associated with type 2 diabetes, obesity, dyslipidemia, alterations in waist-hip ratio, and insulin resistance [4]. It is often called the hepatic manifestation of metabolic syndrome (MS).

Despite the obvious health implications and the economic burden on the healthcare system due to the progressive nature of the disease, there is not a single Food and Drug Administration (FDA) approved drug for the treatment of NAFLD/NASH [5]. Management of NAFLD/NASH is predominantly based on lifestyle modification, decreasing calorie intake, and increasing energy expenditure [6]. The high amount of continuous effort needed to maintain lifestyle modification, exercise, and diet leads to poor compliance with care [7]. Although bariatric surgery has shown efficacy in the management of NASH it is highly invasive and is considered as a last resort in the treatment of NASH.

There are several emerging therapeutic targets in the management of NASH at various stages of development/clinical trials. These range from specific genetic sequences, the alteration of which increases the propensity for the development of NAFLD/NASH, existing drugs that can be repurposed to target NASH treatment, and elements on a molecular level that are essential for the progression of NAFLD to HCC.

This review article aims to identify various therapeutic targets in the treatment of NASH, analyze the mechanism of action of the targets in question, and elaborate on the challenges that can hinder the transition of pharmacological therapy from clinical trials to everyday practice.

## Review

Methodology

**Figure 1:**
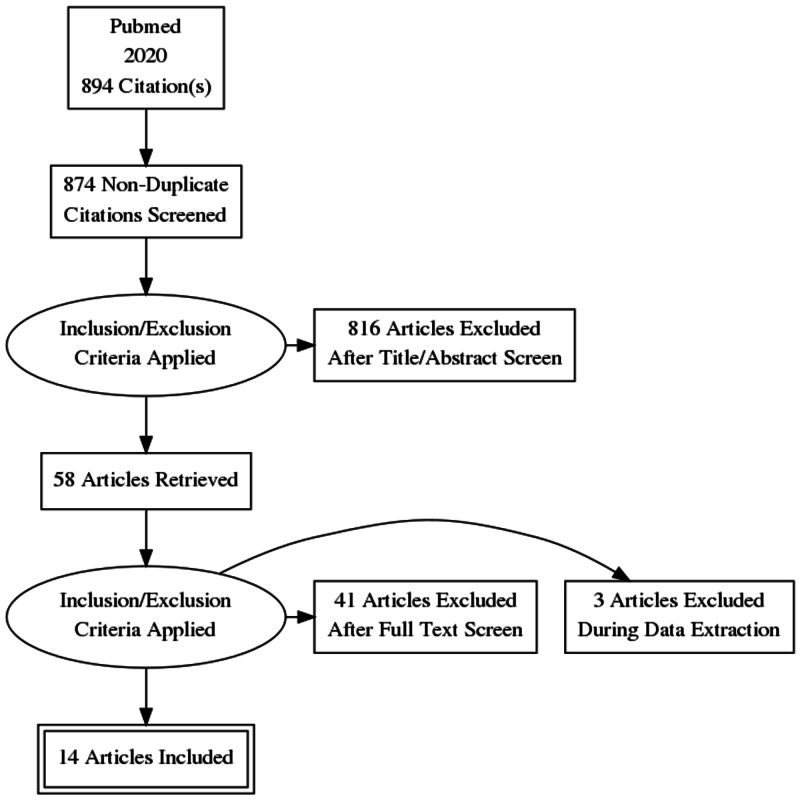
Materials and methods

A comprehensive search strategy was formulated to identify relevant articles. PubMed search was performed using the keywords “NASH” “NAFLD” “molecular targets” “genetic targets” “treatment” “drugs for NASH” “therapeutic targets for NASH” which yielded 894 articles that were published in the last one year. After eliminating duplicates, the articles were further filtered based on the relevance of the abstract to the research question under study. This yielded a total of 16 studies that were included in this review (Figure 1). Inclusion criteria incorporated peer-reviewed articles published in 2020, experimental studies were included, animal studies, and studies published in English. Systematic review studies were excluded from this review article (Figure 1).

Discussion

Molecular Targets

ULK1: Pathogenesis of NASH can be explained by a two-hit hypothesis where the first hit is due to the accumulation of triglycerides within the hepatocytes which lead to steatosis. The second hit is inflammation of the hepatocytes due to various factors like reactive oxygen species excess, increased endoplasmic reticulum stress, gut-derived endotoxins, and the release of proinflammatory cytokinins [8].

Lipotoxic stress due to the presence of saturated fatty acid within the cell drives the cell to apoptosis which is identified as a key pathologic mechanism in NASH.

Unc-51 like autophagy activating kinase 1 (ULK1) is an autophagy activating kinase that is hypothesized to maintain mitochondrial homeostasis. The study conducted by Park et al. demonstrates that ULK1 protects the liver from lipotoxicity by removal of damaged mitochondria via autophagic Kelch-like epichlorohydrin (ECH)-associated protein 1 (KEAP1) degradation and resulting nuclear factor, erythroid 2 like 2 (NFE2L2) activation [9].

In a healthy hepatocyte, KEAP1 interacts with NFE2L2 and suppresses its expression, and promotes the degradation of NFE2L2. When there is increased oxidative stress, it alters the cysteine residues in the KEAP1 thereby leading to activation of NFE2L2. The activation of NFE2L2 leads to the transcription of various genes involved in the autophagy of damaged mitochondria(mitophagy). Sequestosome-1 (SQSTM1/p62) is a receptor protein that promotes autophagy by disrupting the interaction of KEAP1 with NFE2L2 [10,11].

This study demonstrated that ULK1 is a key driver of autophagy as it enhances the interaction between SQSTM1/p62 and KEAP1(9).

**Figure 2:**
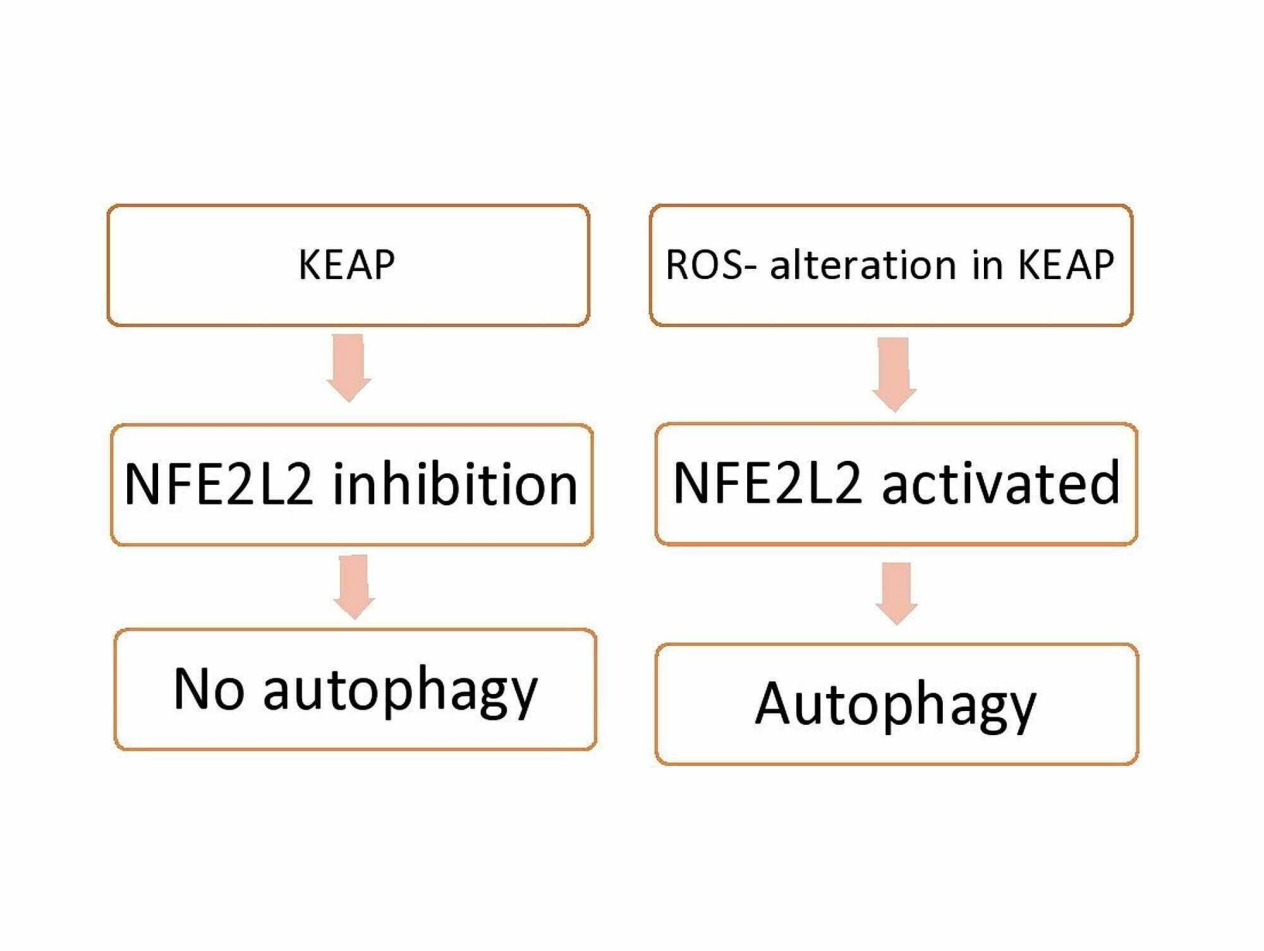
Unc-51 like autophagy activating kinase 1

It has been previously demonstrated that mitophagy happens through the interaction of phosphatase and tensin homolog (PTEN) induced putative kinase 1-parkin RBR E3 ubiquitin-protein ligase (PINK1-PRKN). Damaged mitochondria release PINK1 due to loss of membrane potential. Accumulation of PINK1 outside the mitochondria leads to recruitment of PRKN which leads to mitophagy. Park et al.’s, study also demonstrated that ULK1 promoted the PINK1-PRKN mediated mitophagy by aiding the formation of the SQSTM1-PINK1 complex which further promoted the clearance of damaged mitochondria [9]. This process is represented in Figure 2.

CD36 as a key driver in the pathogenesis of NAFLD: CD36 is a multifunctional signaling molecule whose role in lipid metabolism is to function as a macrophage receptor for LDL (oxidized) and transporter for FFA [12]. Overexpression of CD36 in the liver hepatocyte has been shown to increase lipid accumulation and contribute to the progression of NAFLD [13]. Studies have shown that CD36 is a negative mediator of autophagy thereby contributing to NASH pathogenesis [14]. CD36 also causes hepatic steatosis by acting as a target for various nuclear receptors [15].

**Figure 3:**
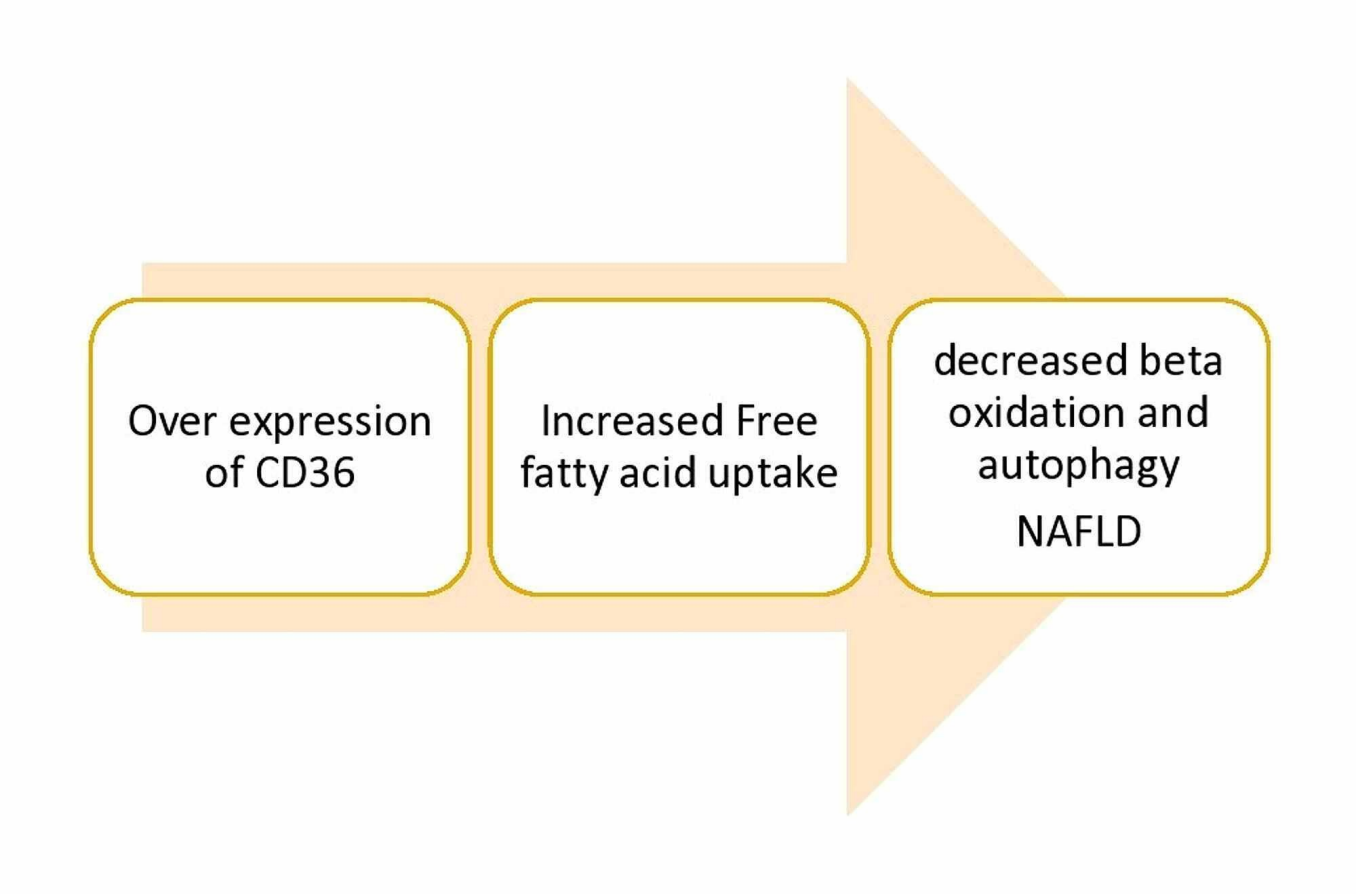
CD36 as a key driver in the pathogenesis of NAFLD NAFLD: non-alcoholic fatty liver disease

Furthermore, a soluble CD36 was found in human plasma and has been hypothesized to be a marker for NAFLD [16]. The study conducted by Rada et al. discussed the role of CD36 as a potential therapeutic target for NAFLD (Figure 3) [17].

CD147 as a potential drug target for NASH: Zhang et al., conducted a study to determine the significance of CD147 in the pathogenesis of NAFLD to NASH progression. Inflammation is a key component of NASH development. CD147 is a membrane glycoprotein that regulates inflammatory responses via NFkB (nuclear factor kappa B) pathway [18]. Their study demonstrated an increased expression of CD147 in mice that had a diet that induces NASH. CD147 is also found to stimulate the NRLP3 inflammasome pathway which is involved in causing cell death in hepatocytes through activation of caspases. Previous studies have brought to light the involvement of Cyclophilin A (Cyp A) an extracellular substance secreted by cells that are inflamed. This Cyp A in turn binds to the surface protein CD147 and causes cell death via the nuclear factor kappa B (NFkB) pathway. This study confirmed this process by demonstrating an elevated level of Cyp A expression NASH models [18]. During this study, it was also noted that Cyp A inhibition in mice hepatocytes lead to an increase in the NRLP3 and NFkB expression. Whereas this was not observed in mice hepatocytes with CD147 deletion. It was also observed that Cyp A inhibitor directly suppressed hepatic lipid accumulation [18]. The study thus provided two potential targets for NASH treat including Cyp A inhibitor and CD147 deletion [18].

Role of L-selectin/CD62L in the pathogenesis of NASH: L-selectin is an adhesion molecule involved in leukocyte recruitment to the site of inflammation. At the site of inflammation, it binds to inducible ligands like mucosal addressin cell adhesion molecule 1. Drescher et al. in their study established that CD62L was detected at a higher level in patients with NASH compared to control subjects. The CD62L messenger ribonucleic acid (mRNA) was expressed at a higher level in patients with NASH when compared to patients with simple steatosis [19]. Flow cytometry also showed a significant increase in the CD62L levels in the intrahepatic immune cells in patients with NASH when compared to control subjects. On performing an experimental study on mice, Drescher et al. found that CD62L deficient mice had less pronounced metabolic syndrome, lower fat content both visceral and subcutaneous, and developed a lower grade of NAFLD with lesser ballooning degeneration and lower serum transaminases compared to wild type mice [19]. His study also demonstrated that there is a reduction in the level of inflammatory cell infiltration in CD62L deficient mice when compared to wild-type mice (WT mice). The anti-inflammatory properties of CD62L deficiency were further consolidated by an increased level of regulatory T cell and lower levels of pro-inflammatory cytokines in CD62L deficient mice [19].

The production of reactive oxygen species is a key driver in the development of NASH. CD62 deficient mice were found to produce significantly lower levels of (reactive oxygen species) ROS compared to wild-type mice thereby strengthening the role of CD62L in the development of NASH [19]. Fibrosis in the liver was noticeably lower in CD62L deficient mice. This can be demonstrated by the less severe activation of stellate cells and lower collagen deposition [19]. CD62L is therefore a potential target in NAFLD/NASH treatment either via gene knockout therapy or with the development of CD62L blocking antibodies. This will act by reducing inflammation and stress due to the production of ROS in damaged hepatocytes [19].

Total flavonoids from Citrus Paradisi cv. Changshanhuyou (TFCH) in preventing oxidative damage: Nuclear factor erythroid 2-related factor (Nrf2) is a signaling molecule that induces transcription of various anti-oxidant genes in response to oxidative stress. The TFCH are experimental compounds derived from flavonoid which have shown to upregulate Nrf2 mediated antioxidant properties. The findings were brought forward by Shi et al., in his article where he experimented with mice and human hepatocytes. Malondialdehyde (MDA) and 8-iso-PGF2a which are the final products of lipid peroxidation, were found to be decreased in TFCH treated NASH models and there was an associated increase in the superoxide dismutase (SOD), glutathione (GSH) which are known to decrease oxidative stress [20].

**Figure 4:**
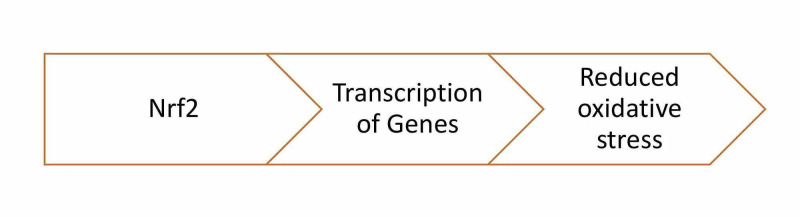
Total flavonoids from Citrus Paradisi cv. Changshanhuyou in preventing oxidative damage

 Triglyceride accumulation and lipotoxicity are considered to the initial step in the pathogenesis of NASH, this can be evidenced by the increased levels of high-density lipoprotein (HDL), low-density lipoprotein (LDL), triglycerides (TG), and free fatty acids (FFA) as well as an increase in liver transaminases released by damaged hepatocyte (Figure 4). There was a significant reversal in the levels of these biomarkers in NASH models treated with TFCH in the study conducted by Shi et al. [20]. 

Protective effect of perilipin 5 in NASH development: Perilipin 5 is a protein that coats the lipid molecule and a key regulator of lipid metabolism. Asimakopoulou et al. conducted a study to determine the significance of perilipin in the pathogenesis of NASH. In this study, comparisons were made between wild-type mice and mice with perilipin deficiency which was both fed a high-fat diet. The study results confirmed that Perilipin deficiency decreased the amount of lipid accumulation within the hepatocytes. This is evidenced by the lower levels of circulating TGs in perilipin deficient mice when compared to wild-type mice. The deficiency of perilipin also reduced the inflammatory action within hepatocytes via the NLR family pyrin domain-containing three (NLRP3) pathway. This can be confirmed by the lower levels of caspases in perilipin deficient mice. Liver enzymes like aspartate aminotransferase (AST) and alanine aminotransferase (ALT) in perilipin deficient mice, when compared to wild-type mice, were significantly reduced. Additionally, the accumulation of inflammatory cells like white blood cells (WBC) were greatly reduced in perilipin deficient mice [21]. Perilipin deficient mice were further found to have lesser steatosis, ballooning degeneration and hepatocyte damage ultimately leading to lesser liver cell death [21].

Pre-Existing Drugs

Zafirlukast: Schierle et al., in their study, derive a compound from zafirlukast that has the ability to interact with farnesoid X receptor and soluble epoxide hydrolase (FXRA/sEHi). The combination of which can prove to be a potential therapeutic target in the treatment of metabolic syndrome and NASH [22]. The farnesoid X receptor when activated has the ability to decrease hepatic steatosis and fibrosis which has been implicated in the development of NASH as one of the key pathological changes. This activity of NASH is brought about by Zafirlukast-derived FXRA/sEHi 13 and 16 [22]. The sEH in turn has exhibited a strong anti-inflammatory effect which can be the turning point in the treatment of NASH. The combination of these two unrelated mechanisms can therefore be a potential target in treating the raising global epidemic [22].

Febuxostat: Febuxostat is a xanthine oxidase inhibitor that has been shown to slow the progression of NAFLD/NASH. Nishikawa et al. conducted a study to test the efficacy of febuxostat in mice with NASH which was then followed by a single-arm, open-label trial of febuxostat in patients with NASH who also have hyperuricemia [23].

Mice were fed a high-fat diet, febuxostat was shown to decrease the xanthine oxidase level in the hepatocytes of these mice, lower insulin resistance, improve glucose tolerance, prevent lipid accumulation and steatosis, protect against hepatic fibrosis. The hepatic damage due to inflammation and oxidative stress was also lower in mice treated with febuxostat [23].

In patients with hyperuricemia treated with febuxostat, there was a significant decrease in the serum uric acid levels and lower LDH. In patients who had a moderate liver injury the liver enzymes like AST, ALT was lowered following treatment with febuxostat. Liver biopsies of these patients showed a significant improvement in steatosis but no change in the inflammation or ballooning degeneration [23].

Saroglitazar: Saroglitazar is a Peroxisome proliferator-activated receptors (PPAR) alpha and gamma agonist which was formulated for the treatment of dyslipidemia. Kumar et al. conducted a study on mice that were fed a high-fat diet and sugar water. Compared to mice without any intervention, mice that were given saroglitazar showed significant changes [24].

It was shown that saroglitazar acted through the PPAR alpha and gamma pathways. Mice that were fed saroglitazar showed a decrease in insulin resistance - reduced fasting blood sugar, and obesity. Saroglitazar also decreased the circulating levels of AST, ALT, and tumor necrosis factor (TNF) alpha and increased adiponectin which points towards a protective role in liver injury. Saroglitazar was shown to reduce circulating levels of triglyceride and total cholesterol without affecting HDL cholesterol [24].

Histological analysis of saroglitazar treated mice hepatocytes showed no ballooning degeneration, decrease in steatohepatitis, and lower mean fibrosis when compared to control mice. Saroglitazar was also found to upregulate Nrf2 gene transcription which is indicated to have a protective effect against oxidative stress. Saroglitazar was found to modulate metabolic pathways and several genes involved in immune response, phagocytosis, and inflammation [24].

Anagliptin: In recent years, numerous studies were published which have shown the efficacy of anti-diabetic drugs like sodium-glucose co-transporter-2 (SGLT2) and dipeptidyl peptidase-4 (DPP4) inhibitors in preventing the progression of NAFLD to NASH [25-27].

In the study conducted by Kawakubo et al., genetically obese mice on a western diet are treated with anagliptin. The effect of anagliptin on the progression of NASH was observed. The study showed that anagliptin reduced ballooning degeneration, inflammation, and fibrosis thereby preventing the progression of NAFLD to NASH [28].

Long-term treatment with anagliptin also greatly decreased tumorigenesis in the liver hepatocytes and brought about a reduction in tumor size. Anagliptin worked at the molecular level by preventing the upregulation of genes involved in tumor formation [28].

Liraglutide: Glucagon-like peptide 1 (GLP1) is an incretin secreted by the body in response to food which exerts hypoglycemic effects. Hypoglycemic effects are the result of increased insulin secretion from islet cells, decreased glucagon secretion, appetite suppression, and early satiety [29].

Recent studies have shown GLP1 agonists like Liraglutide to affect the renin-angiotensin system (RAS), RAS has been associated with metabolic syndrome due to its effect on glycolipid metabolism and insulin sensitivity [30].

In the study conducted by Yang et al., the effect of GLP1 agonist liraglutide on the hepatic RAS was explored. Liraglutide was found to regulate hepatic steatosis by dual pathway, acting on both ACE/AngII/AT1R and ACE2/Ang1-7/Mas. In angiotensin-converting enzyme 2 (ACE2) knockout mice, the hepatic local RAS was overactivated liraglutide upregulated ACE2 activity. Liraglutide was also found to downregulate ACE activity. This led to the suppression of gluconeogenesis and inflammation in the liver thereby preventing NAFLD progression [31].

Cenicriviroc: Cenicriviroc is a newer oral anti-CC-motif chemokine receptor 2/5 (CCR2/5). CCR2/5 is seen on circulating monocyte/macrophages and hepatic stellate cells. It is hypothesized that the activation of these receptors leads to inflammation and fibrosis in the liver contributing to NASH pathogenesis [32].

Soto and Lim discuss the ongoing clinical trials testing the efficacy and safety profile of this drug, at present the Phase 2a clinical trials are complete whereas the Phase 2b and Phase 3 are expected to be completed in late 2020 and 2021, respectively. According to phase 2a the drug was found to improve hepatic fibrosis by 1 stage without worsening the steatohepatitis. This improvement in fibrosis was found to be greater in patients with advances in NASH scoring [33].

Phase 2b trials are being conducted to find the combined efficacy of cenicriviroc and tropifexor (TXR) which is a non-bile acid farnesoid X receptor agonist [33]. The summary of all the drugs and their stage of development is given in Table 1.

**Table 1:** Comprehensive data on the various pre-existing drugs available for the treatment of NASH NASH: non-alcoholic steatohepatitis; PPAR α/γ: peroxisome proliferator-activated receptors alpha/gamma; GLP1 : glucagon-like peptide 1; DPP4: dipeptidyl peptidase 2

Study	Author	Drug	Mechanism of action	Outcome	Phase of trial
Dual farnesoid X receptor/soluble epoxide hydrolase modulators derived from zafirlukast	Schierle et al. [22]	Zafirlukast	Interaction with farnesoid X receptor and soluble epoxide hydrolase	Reduction in hepatic steatosis, fibrosis, and inflammation	Preclinical
Xanthine oxidase inhibition attenuates insulin resistance and diet-induced steatohepatitis in mice	Nishikawa et al. [23]	Febuxostat	Xanthine oxidase inhibitor	prevent lipid accumulation and steatosis, protect against hepatic fibrosis	Preclinical
The PPAR α/γ agonist saroglitazar improves insulin resistance and steatohepatitis in a diet-induced animal model of non-alcoholic fatty liver disease	Kumar et al. [24]	Saroglitazar	PPAR alpha and gamma agonist	Protective against oxidative stress and hepatic injury	Preclinical
Dipeptidyl peptidase-4 inhibition prevents non-alcoholic steatohepatitis–associated liver fibrosis and tumor development in mice independently of its antidiabetic effects	Kawakubo et al. [28]	Anagliptin	DPP4 inhibitor	reduced ballooning degeneration, inflammation, and fibrosis. Decreased tumerogenesis	Preclinical
Liraglutide attenuates non-alcoholic fatty liver disease in mice by regulating the local renin-angiotensin system	Yang et al. [31]	Liraglutide	GLP1 agonist	Regulate steatosis and prevent inflammation	Preclinical
Evaluating the therapeutic potential of cenicriviroc in the treatment of non-alcoholic steatohepatitis with fibrosis: a brief report on emerging data	Soto and Lim [33]	Cencriviroc	Oral anti CCR2/5	Improve hepatic fibrosis	Phase 3

Gene Therapy

MBOAT 7: Vast number of genes are found to be associated with the pathogenesis of NAFLD progression through the human genetic association studies [34]. Membrane-bound O-acyl transferase7 (MBOAT7) is one such gene and the specific variant MBOAT7 rs641738T is associated with the progression of alcoholic liver disease, NAFLD, and hepatitis B and C [35]. MBOAT7 is a transmembrane protein that is involved in the PI side-chain remodeling within the hepatocytes. MBOAT7 deficiency is found to produce fibrosis in the liver [36].

Thangapandi et al. conducted a study with both biopsied human liver cells from NAFLD patients and murine liver tissue. His study demonstrated that the MBOAT7 rs641738T variant is associated with hepatic steatosis as witnessed by the increase in cholesterol esters in hepatocytes. He was also able to demonstrate an upregulation of lipogenic enzymes with downregulation of genes involved in fatty acid oxidation [37]. This study further established that the mechanism of fibrosis was independent of inflammation and was mainly mediated through the hepatic stellate cells and myofibroblast [37].

MCJ silencing: Beta oxidation of fatty acid in the liver produces NADH and FADH2 which when accumulated in the hepatocyte can suppress beta-oxidation through negative feedback. The electron transport chain uses up the NADH and FADH2 thereby prevents the accumulation of lipids in the hepatocytes. Methylation controlled J (MCJ) protein is a negative regulator of the electron transport chain (ETC) and inhibits the complex I of ETC [38].

MCJ has been identified as a target to prevent the progression of NASH. Inhibition of hepatic MCJ helps alleviates the inhibitory effect on ETC and prevents lipid accumulation. However increased beta-oxidation was associated with the accumulation of ROS which can lead to NAFLD progression, however, the activation of ETC diverts the ROS into the production of respiratory complexes reducing the damage caused due to ROS [39].

Barbier-Torres et al. designed a small interfering ribonucleic acid (SiRNA) and a liver-specific delivery system to silence the MCJ and demonstrate the above-mentioned findings. His study also demonstrated that MCJ levels are higher in the hepatocytes of patients with NAFLD compared to healthy patients [39].

Laboratory to Bedside Transition

Although non-alcoholic fatty liver disease is a globally prevalent and rapidly growing epidemic there is no FDA-approved drug for its treatment. This article discussed the various modalities of therapeutics that are at various stages of development. However, a question arises as to when would these experimental drugs make it to bedside treatment. Many of the molecular and genetic targets discussed in this review article are in animal trials or human cell line trials (preclinical). It can take multiple years for these preclinical trials to progress to clinical trials where their safety and efficacy are documented. The duration of the clinical trials is also highly variable and dependent on factors like availability of infrastructure, patients, and funding to name a few. Even though drugs like liraglutide and anagliptin are already prevalent in the market they are yet to undergo clinical trials that identify the therapeutic dose needed for the treatment of NAFLD and the adverse effects that develop at that dose of the medication. Genetic and molecular studies using gene knockout or knock-in and targeted delivery are relatively newer techniques and might not be as widely accepted by the general public.

Limitations

This study only included articles that were published in 2020 in the English language. PubMed was the only database used to gather the articles. Hence, this study does not represent all of the available literature about the topic at hand.

## Conclusions

Fatty liver disease is a diagnosis of middle-aged men and women with a prevalence of 25% worldwide. The untreated or inadequately treated fatty liver disease could progress to cirrhosis, end-stage renal disease, and hepatocellular carcinoma. This study provided a comprehensive list of drugs and molecular or genetic techniques that are in development for the treatment of fatty liver disease. The mechanism through which these drugs work and the predicted outcomes were discussed. The study also addressed the factors that could potentially hinder the transfer of these experimental studies into everyday practice.
